# Editorial: Understanding Altered Muscle Activation After Central or Peripheral Neuromuscular Injuries

**DOI:** 10.3389/fneur.2021.642207

**Published:** 2021-07-29

**Authors:** Francesco Negro, Xiaogang Hu, Jun Yao

**Affiliations:** ^1^Department of Clinical and Experimental Sciences, Research Centre for Neuromuscular Function and Adapted Physical Activity “Teresa Camplani”, Università degli Studi di Brescia, Brescia, Italy; ^2^Joint Department of Biomedical Engineering, University of North Carolina at Chapel Hill, North Carolina State University, Chapel Hill, NC, United States; ^3^Department of Physical Therapy and Human Movement Sciences, Northwestern University, Chicago, IL, United States; ^4^Northwestern University Interdepartmental Neuroscience, Northwestern University, Chicago, IL, United States; ^5^Department of Biomedical Engineering, Northwestern University, Evanston, IL, United States

**Keywords:** motor impairment, pathophysiology, motor unit, rehabilitation, electromyogram

Appropriate muscle activation plays a critical role in our daily motor activities. Therefore, quantification of muscle activation becomes an important way for diagnosing or assessing neuromuscular conditions, interfacing with machines, and assessing the efficacy of rehabilitation or assistive strategies. Recent developments in quantifying the neural control of muscles have provided an unprecedented possibility to probe into the nervous system for understanding motor impairment in pathological conditions. This Research Topic collected 15 articles that cover a wide range of novel methods or findings of muscle activation in various basic-scientific or clinical research following central or peripheral neuromuscular injuries.

In individuals with a neurological or neuromuscular condition such as a stroke or multiple sclerosis, commonly-reported movement deficits include muscle weakness, spasticity, and discoordination. Electromyography (EMG) is a non-invasive and easy-to-use probe for investigating the central nervous system's control of movements and peripheral muscular properties. It has been widely used in pathologic and rehabilitation studies. To access the stroke-induced weakness, Son and Rymer quantified the EMG-torque slops during isometric plantarflexion contractions with different ankle angles. They also used ultrasound to measure the muscle thickness and tissue stiffness. Their results reflect the reduced efficiency in muscular contraction following a stroke, which may be linked to the increased tissue stiffness. To understand the mechanisms underlying post-stroke spasticity and investigate its new treatment, Lu et al. measured the intramuscular needle EMGs before and immediately after dry needling to the flexor digitorum superficialis in stroke individuals with spasticity in finger flexors. Their preliminary results showed that dry needling leads to immediate spasticity reduction, suggesting that latent trigger points possibly exist in spastic muscles. To explore neural mechanisms underlying post-stroke discoordination, Guo et al. quantified corticomuscular coherence based on EEG and EMG recordings. Based on results from 14 stroke participants and 10 age-matched controls, they concluded that the post-stroke proximal muscular compensations from the elbow to finger movements were cortically originated. Also using the coherence method, Laine and Valero-Cuevas reported amplified intermuscular coherence in the alpha-band in individuals with Parkinson's disease compared with healthy control participants, suggesting that intermuscular coherence in the alpha-band can be an efficient biomarker for detecting the presence of Parkinson disease. Volitional control of the ankle dorsiflexors is commonly impaired after a spinal cord injury (SCI). Hope et al. reviewed the potential association between neurophysiological changes at the corticospinal and spinal levels with impaired volitional ankle control and spasticity after SCI. This review evaluated how changes in corticospinal transmission and spinal reflex excitability contribute to volitional ankle control and spasticity. Patients with multiple sclerosis can markedly reduce push-off and toe-clearance during gait compared to healthy individuals. Jonsdottir et al. evaluated whether altered neuromotor control, as represented by muscle synergies, improves with rehabilitation. This study investigated changes in ankle motor control and associated biomechanical parameters after a 20-session intensive treadmill training. The results showed a reorganization of distal leg muscle synergy related to neurophysiological changes induced by rehabilitation, which was associated with improved ankle performance and gait speed.

In recent years, the use of multichannel intramuscular and surface electromyographic recordings offers the potential to separate central and peripheral contributions of muscle activation, and therefore unravel unique features of neuromuscular diseases. Wilson et al. used intramuscular EMG decomposition to quantify discharge rate statistics of individual motor units in patients suffering from mild-to-moderate Parkinson's disease. Interestingly, the results showed that variability in motor unit discharge and elbow flexion/extension forces were the main factors that may contribute to motor dysfunction in this patient population. Other neuromuscular diseases can also exhibit distinctive modifications in the central and peripheral properties of individual motor units. Negro et al. employed motor unit decomposition and tracking from high-density surface EMG recordings to show disease-related central changes in a cohort of chronic stroke individuals after a fatigue task. The study demonstrated that fatigability in the affected side of stroke individuals is associated with impaired rate coding and recruitment of motor units. Similarly, Vieira et al. demonstrated that neural plasticity in stroke survivors could modify the peripheral properties of motor units in the medial gastrocnemius muscles. Specifically, the motor unit action potentials in the paretic muscles showed a more extensive distribution than the non-paretic side not explained by variations in anatomical factors (subcutaneous thickness), leading to a new generation of markers of neuromuscular plasticity in stroke individuals. Motor Unit Number Index (MUNIX) has been accepted as a neurological tool for indexing the number of functioning motor unit (MU) of the target muscle, which provides a susceptive biomarker for innervation conditions in patients with neurodegenerative diseases. Gao et al. examined the effect of channel number and channel location on the repeatability of MUNIX. High-density surface electromyogram (EMG) signals were recorded from the biceps brachii muscles. The study found a significantly improved repeatability (quantified by the coefficient of variation across trials) with the proposed multi-channel MUNIX approach. Higher variability of single-channel MUNIX was observed when the recording channel is positioned away from the innervation zone. This study identified potential sources of MUNIX variations, and provided novel perspectives to improve the repeatability.

Interestingly, EMG-based electrophysiological tools may also be used to assess the neural changes in motor disorders after neural stimulation interventions. Gogeascoechea et al. assessed modifications in the common synaptic oscillations in the neural drive to muscles after the applications of trans-spinal direct current stimulation in a cohort of spinal cord injury patients. Impressively, the estimated common synaptic input decreased after administering the stimulation protocol, and the effect persisted for tens of minutes. Repetitive peripheralmagnetic nerve stimulation (rPMS) is a putative adjuvant therapy that improves the mobility of patients with spasticity, Zschorlich et al. investigated the underlying mechanisms of rPMS in spasticity reduction. The study found that, after a 5-min rPMS, there is a significant reduction of tendon-reflex activity of the targeted muscle. The findings indicate that rPMS could be used as an adjuvant therapy to reduce spasticity during motor rehabilitation of locomotion or postural control. Amyotrophic lateral sclerosis (ALS) is a type of motor neuron disease that leads to progressive muscle atrophy and weakness. Shang et al. identified the characteristics of repetitive nerve stimulation (RNS) in patients with ALS, and further verified the electrophysiological exclusion criteria of ALS. The study compared the amplitudes of the compound muscle action potential (CMAP) between the trapezius muscle and the abductor digiti minimi in low-frequency RNS. CMAP decremental responses in RNS were common in ALS patients, suggesting abnormalities of neuromuscular junctions. The study also suggested the possibility to consider a decrement >20% in CMAP from RNS as a diagnostic exclusion criterion for ALS.

Lastly, EMGs are also widely used to interface with machines for rehabilitation purposes. Kadone et al. reported a case result in a patient with Thoracic Myelopathy due to ossification of the posterior longitudinal ligament. An EMG controlled Hybrid Assistive Limb was used to improve this patient's walking ability. Both kinematic and Kinetic data before and after a 10-session intervention were reported to demonstrate the post-intervention gate improvement and the altered central nervous system control for relative muscles, suggesting that an EMG-control rehabilitation tool can effectively result in functional regain. The combination of neural decoding and the application of therapeutic interventions may open up new opportunities to design real-time closed-loop control systems for rehabilitative applications. Accordingly, Nizamis et al. demonstrated the use of high-density surface (HD-sEMG) recordings to estimate the spatial distribution of HD-sEMG patterns in the forearm of patients suffering Duchenne muscular dystrophy for the future control of robotic exoskeletons. The results of this study showed unequivocally that patients, even with clear progressive alterations in hand/wrist motor control, can produce repeatable spatial muscle activation patterns, and they can be used for the myocontrol of wearable exoskeletons.

## Author Contributions

All authors listed have made a substantial, direct and intellectual contribution to the work, and approved it for publication.

## Conflict of Interest

The authors declare that the research was conducted in the absence of any commercial or financial relationships that could be construed as a potential conflict of interest.

## Publisher's Note

All claims expressed in this article are solely those of the authors and do not necessarily represent those of their affiliated organizations, or those of the publisher, the editors and the reviewers. Any product that may be evaluated in this article, or claim that may be made by its manufacturer, is not guaranteed or endorsed by the publisher.

